# A Comprehensive Review of Nanoparticles in the Fight Against Antimicrobial Resistance

**DOI:** 10.3390/pathogens14111090

**Published:** 2025-10-26

**Authors:** Antonios Mouzakis, Periklis Panagopoulos, Dimitrios Papazoglou, Vasileios Petrakis

**Affiliations:** 1 Laboratory of Molecular Immunology, Department of Molecular Biology and Genetics, Democritus University of Thrace, 68100 Alexandroupolis, Greece; antonios.n.mouzakis@gmail.com; 2 Second Department of Internal Medicine, University General Hospital Alexandroupolis, Democritus University of Thrace, 68100 Alexandroupolis, Greece; ppanago@med.duth.gr (P.P.); dpapazog@med.duth.gr (D.P.)

**Keywords:** antimicrobial resistance, nanoparticles, nanotoxoid vaccines

## Abstract

(1) Background: The escalating crisis of multidrug-resistant (MDR) bacteria presents a formidable threat to global public health, necessitating the urgent development of alternative antimicrobial strategies. Nanoparticles (NPs) have emerged as a promising frontier in this effort, leveraging their unique physicochemical properties and multi-modal mechanisms of action to combat bacterial infections. This systematic review aims to comprehensively evaluate the current body of evidence on the dynamic interplay between nanoparticles and bacterial resistance. (2) Methods: A comprehensive search of electronic databases, including PubMed, Scopus, and Web of Science, was performed using a combination of keywords and Medical Subject Headings (MeSH) terms to identify relevant primary research articles. Eligibility criteria focused on studies evaluating the antimicrobial effects of nanoparticles on MDR bacterial strains, reporting on mechanisms of action, efficacy, or resistance development. (3) Results: The synthesis of findings revealed that nanoparticles exert their antimicrobial effects through multiple pathways, including the generation of reactive oxygen species (ROS), direct disruption of bacterial membranes, and the release of toxic ions. However, the analysis also confirmed that bacteria have evolved sophisticated defense mechanisms against nanoparticles, including surface modifications that prevent adhesion, upregulation of efflux pumps, and chemical neutralization of toxic ions. (4) Conclusions: Nanoparticles represent a potent and versatile tool in the global effort to combat antimicrobial resistance. Their long-term efficacy is not guaranteed, as bacteria have shown a remarkable capacity for adaptation. The future of this field lies in the development of rationally designed nanoparticle systems that not only possess intrinsic antimicrobial activity but also actively disarm bacterial resistance mechanisms. This includes the strategic use of synergistic combinations with conventional antibiotics and the exploration of resistance-agnostic approaches like nanotoxoid vaccines.

## 1. Introduction

Antimicrobial resistance (AMR) is a profound and escalating threat to global public health, representing a critical challenge that has outpaced the development of new therapeutic agents. The crisis is driven by the rapid evolution of resistance mechanisms in pathogens and the limited discovery of effective, next-generation antibiotics [1]. The gravity of this challenge is underscored by sobering statistics, with estimates indicating that antibiotic resistance is already responsible for more than one million deaths annually worldwide [1,2]. Without decisive action, this number is projected to rise dramatically.

The limitations of conventional antibiotics are at the heart of this crisis. Traditional small-molecule drugs are often designed to target a single biochemical pathway or a specific molecular structure within a bacterium [3]. This precise, ‘single-target’ mechanism of action makes them vulnerable to a single point of failure. A single genetic mutation can confer resistance, rendering an entire class of drugs ineffective. Furthermore, many conventional therapies are constrained by their fixed chemical structures, limiting their ability to penetrate complex bacterial communities, such as biofilms, or to reach intracellular pathogens sequestered within host cells [4]. These fundamental shortcomings have fueled the widespread emergence of ‘superbugs’ and multidrug-resistant (MDR) bacterial strains, against which existing treatments are often powerless [1]. In this context, nanotechnology has emerged as a promising frontier offering innovative solutions to address the limitations of conventional therapies. Nanoparticles (NPs), defined as materials ranging from 1 to 100 nanometers, possess unique physicochemical properties that distinguish them from their bulk counterparts [5]. Their high surface-to-volume ratio, tunable surface chemistry, and multifunctionality enable them to interact with biological systems in novel ways [6]. The small size of NPs allows them to bypass many of the barriers that conventional drugs cannot, while their surface can be engineered to perform multiple functions simultaneously, from drug delivery to direct bactericidal action [1]. This represents a fundamental shift in the approach to antimicrobial therapy- from a specific, single-point attack to a broad, multi-pronged assault that is inherently more difficult for bacteria to counteract.

The existing literature on nanoparticles and antimicrobial resistance is vast but fragmented. Studies often focus on a single nanoparticle type, a specific bacterial strain, or an isolated mechanism of action. Therefore, a systematic, comprehensive synthesis of this scattered evidence is essential to consolidate findings, identify critical knowledge gaps, and provide a clear, evidence-based roadmap for future research and clinical translation. This review directly addresses these critical needs and will systematically explore the transformative role of nanoparticles, from their intrinsic antimicrobial properties and advanced therapeutic applications to the critical challenges that must be addressed for their successful clinical translation.

## 2. Materials and Methods

### 2.1. Protocol and Registration

This systematic review was conducted strictly according to a pre-registered protocol to ensure methodological rigor and transparency and prevent selective reporting. The review process followed the guidelines outlined in the PRISMA 2020 statement for systematic reviews [7].

### 2.2. Eligibility Criteria

Primary research articles were screened for eligibility based on predefined inclusion and exclusion criteria. Primary research articles, including in vitro studies, animal models, and clinical trials (if available), published in peer-reviewed journals, studies involving bacterial pathogens, particularly those identified as multidrug-resistant or possessing known mechanisms of antibiotic resistance, studies evaluating any form of nanoparticle or nanomaterial with antimicrobial properties, including metallic, polymeric, and carbon-based nanomaterials and studies reporting on antimicrobial efficacy (e.g., minimum inhibitory concentration (MIC), zone of inhibition (ZOI)), mechanisms of action, or bacterial resistance/adaptation mechanisms against nanoparticles were included. Non-primary research, such as reviews, editorials, commentaries, and book chapters, not focused on the antimicrobial effects of nanoparticles and studies lacking a clear description of the methodology, nanoparticle characteristics, or key outcomes were excluded.

### 2.3. Information Sources and Search Strategy

A comprehensive electronic search was conducted across three major databases: PubMed, Scopus, and Web of Science. These databases were selected for their extensive coverage of biomedical, engineering, and materials science literature, providing a holistic view of the subject [8]. The search strategy employed a combination of keywords and Medical Subject Headings (MeSH) terms to capture the full breadth of relevant literature (“Nanoparticles” OR “Nanostructures” OR “Nanomaterials” OR nanotherapeutics) AND (“Drug Resistance, Microbial” OR “Antimicrobial Resistance” OR “Biofilms” OR “efflux pumps” OR “bacterial resistance”). Additionally, manual searches of the reference lists of all included studies were performed to identify any further relevant articles. The searches were conducted continuously until a final date to ensure the most current data was captured.

### 2.4. Study Selection Process

The study selection process was carried out in two phases to minimize bias and ensure a robust review. In the first phase, the titles and abstracts of all identified records against the eligibility criteria were screened. Studies deemed potentially relevant were moved to the second phase, which involved a full-text review.

### 2.5. Data Extraction

Data were extracted from each included article using a standardized, pre-designed data extraction form. The form was tailored to capture critical information relevant to the research question. The key data points extracted included: study ID, author, year, study design, the specific nanoparticle type (e.g., silver, gold, polymeric, carbon-based), its physicochemical properties (size, shape, surface charge, concentration), the bacterial strain(s) tested (e.g., *E. coli*, *S. aureus*, MRSA), the primary antimicrobial mechanism(s) of the nanoparticle, and any reported bacterial resistance or adaptation mechanism(s). A crucial aspect of this data extraction was the recognition that the efficacy of nanoparticles is not solely dependent on their material composition but is intricately linked to their physicochemical properties. A simple data extraction table listing only the “nanoparticle type” would fail to capture this critical nuance. The review, therefore, collected granular data on NP characteristics and synthesis methods to assess how these variables influence outcomes. This detailed approach exposed a significant challenge in the current literature: the lack of standardized reporting on nanoparticle formulations and experimental protocols. This variability makes direct comparisons between studies difficult and underscores a critical gap that must be addressed for the field to advance.

### 2.6. Risk of Bias Assessment

The methodological quality and potential for bias in the included studies were assessed independently. For randomized controlled trials (RCTs), if any were identified, the RoB 2 tool was used [9]. The ROBINS-I tool was applied for non-randomized studies, such as in vitro experiments or animal models [10]. The assessment was performed across relevant domains, including confounding, selection of participants, classification of interventions, and measurement of outcomes.

### 2.7. Data Synthesis

Given the anticipated heterogeneity of the included studies, which utilized a wide range of nanoparticle compositions, bacterial strains, and experimental conditions, a meta-analysis was not deemed appropriate. Instead, a narrative synthesis approach was conducted to group studies systematically and identify common themes and trends. This qualitative approach allowed for a comprehensive discussion of nanoparticle mechanisms, bacterial resistance pathways, and the challenges of clinical translation. Heterogeneity was a central component of this analysis, with patterns explored based on nanoparticle type, physicochemical properties, and targeted bacterial defense mechanisms. As this review employed a narrative synthesis approach, limited interpretive commentary has been intentionally included within this section to contextualize the thematic patterns emerging from the reviewed studies. This integration is consistent with narrative synthesis methodology, which emphasizes the combination of descriptive and interpretive elements to provide a holistic understanding of the evidence base. Therefore, the synthesis not only categorizes findings but also highlights the evolving conceptual trajectory of nanoparticle research in combating antimicrobial resistance. The evidence revealed that the future of nanomedicine in combating AMR is not about replacing antibiotics, but about a synergistic revolution. Nanoparticles can act as “resistance bypass agents,” actively disarming bacterial defenses, such as biofilm matrices or efflux pumps, while a conventional antibiotic delivers the killing blow. This dual-action approach represents a significant leap forward from simple combination therapies and was a key theme to highlight in the final synthesis [11,12,13].

## 3. Intrinsic Antimicrobial Mechanisms of Nanoparticles

Nanoparticles, particularly those composed of metals and carbon, possess intrinsic antimicrobial properties that do not rely on a loaded drug payload. Their efficacy stems from a diverse and multifaceted array of mechanisms that can simultaneously disrupt multiple bacterial processes (Table 1). This is a significant departure from the single-target approach of conventional antibiotics, which explains why nanoparticles are a compelling alternative [14].

The primary mechanisms of NP-induced cell death can be categorized into three main pathways. The first and most widely documented is the generation of reactive oxygen species (ROS) [21]. Many NPs, especially metal-based ones like silver, zinc oxide, and titanium oxide, can induce oxidative stress within bacterial cells by generating ROS, including hydroxyl radicals and superoxide anions [3]. This oxidative damage is non-specific and widespread, affecting essential cellular components such as lipids, proteins, and DNA, ultimately leading to cell death [3,21].

A second key mechanism is the direct disruption of the bacterial cell membrane [22]. Cationic polymeric nanoparticles, for instance, can interact electrostatically with the negatively charged surface of bacterial membranes, causing structural damage, increasing membrane permeability, and leading to the leakage of intracellular contents [23]. Similarly, carbon-based nanomaterials can physically puncture or stretch the membrane, causing irreparable damage [24]. Finally, many metallic NPs achieve their effect through the controlled release of toxic ions [25,26,27,28]. Silver nanoparticles, for example, release silver ions (Ag^+^) that can bind to the thiol (-SH) groups in bacterial proteins and enzymes, disrupting critical functions like respiration, ATP production, and DNA replication [10].

Different classes of materials exhibit these mechanisms to varying degrees, each with its own unique properties. Silver nanoparticles (AgNPs) have a long history as antimicrobial agents, with documented use dating back centuries [27]. However, the modern innovation lies in engineering silver at the nanoscale, which provides a high surface-area-to-volume ratio that enhances its efficacy even at low concentrations [29]. This allows for a more efficient and targeted use of a known antimicrobial, circumventing the limitations of its bulk form. Gold nanoparticles (AuNPs), while often considered more biocompatible and less toxic, also exhibit antibacterial activity through membrane disruption, ROS generation, and enzyme inhibition [30,31]. Their unique optical properties also make them suitable for photothermal therapy (PPT), where they convert light into heat to ablate bacterial cells [32].

Carbon-based nanomaterials (CBNs) offer a different set of mechanisms. Graphene and its derivatives, like graphene oxide (GO), primarily exert their antibacterial effects through physical interactions [33]. The sharp edges of these materials can physically puncture bacterial membranes; a mechanism often referred to as the “sharp-edge effect” [18,34,35]. This is complemented by chemical mechanisms such as oxidative stress and electron transfer [36]. Similarly, carbon nanotubes (CNTs) induce mechanical damage to cell walls and generate oxidative stress, with their efficacy influenced by factors like size and length [37]. Fullerenes, another form of carbon, can function as photosensitizers, generating ROS upon exposure to light [38,39,40]. The diversity of these mechanisms means that a bacterium that has evolved a defence against one type of nanoparticle, such as an upregulation of antioxidant responses to combat silver-induced oxidative stress, would not be protected against the physical disruption caused by a graphene sheet [40]. This multiplicity of mechanisms is a key reason why nanoparticles are less likely to induce widespread resistance compared to conventional drugs [41].

In addition to these well-documented mechanisms, nanoparticles can also interact with bacteria through a range of surface-level and intracellular processes. They can attach to bacterial surfaces via electrostatic, hydrophobic, van der Waals, and receptor–ligand interactions or penetrate the bacterial membrane and accumulate inside the cell. Some NPs can physically kill bacteria through mechanical stretching and rupture of the cell membrane. After attachment, they can interfere with electron, ion, and nutrient transport, disrupt respiratory function and efflux pumps, or tear the membrane, leading to cellular leakage and death [42].

Once internalized, nanoparticles can induce oxidative and nitrosative stress, leading to the production of reactive oxygen and nitrogen species that damage DNA, alter gene expression, and inactivate crucial proteins and enzymes involved in key signaling pathways. They can also release toxic ions capable of killing bacteria even without direct contact. The antibacterial activity of NPs depends on their physicochemical characteristics—such as size, shape, surface chemistry, and degree of hydrophobicity or hydrophilicity—as well as their concentration and exposure duration. Similarly, bacterial features including species, growth rate, cell wall composition, genetic background, and adaptability determine their susceptibility or resistance to nanoparticles [42].

**Figure 1:**
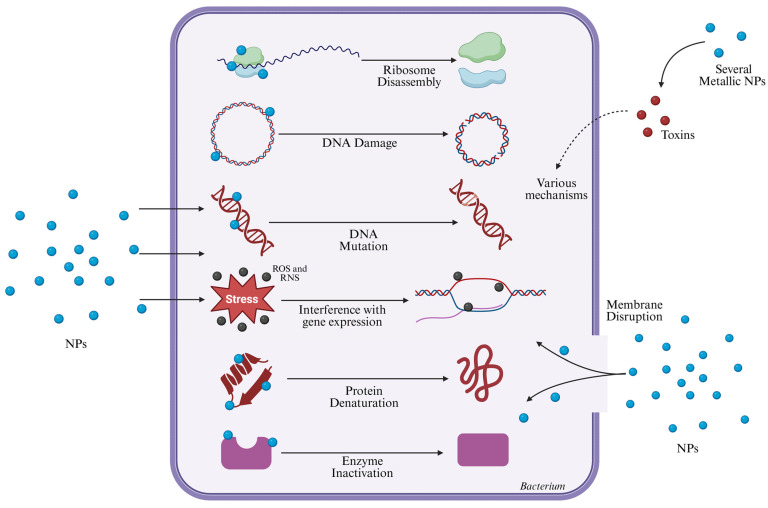
Schematic representation of the antibacterial mechanisms of NPs. NPs attach to bacterial surfaces or penetrate cells, inducing membrane disruption and oxidative/nitrosative stress through reactive oxygen and nitrogen species (ROS and RNS). These processes cause DNA damage and mutation, interfere with gene expression, and lead to protein denaturation and enzyme inactivation. Metallic NPs can also release toxic ions that disrupt essential cellular functions. Together, these multifaceted mechanisms result in bacterial cell death. Adapted from [42]. Created with Biorender.

All of the mechanisms described above can be summarized in Figure 1.

## 4. Advanced Nanoparticle Strategies Against Bacterial Infection

Beyond their intrinsic antimicrobial properties, nanoparticles are increasingly being utilized as sophisticated therapeutic platforms to overcome the complex challenges of bacterial infections, including those related to biofilms and intracellular pathogens. These advanced strategies showcase the true versatility of nanomedicine. One of the most widely explored applications is their use as targeted drug delivery systems. Nanoparticles can act as ‘smart carriers’ for conventional antibiotics, improving their efficacy and reducing systemic toxicity [43,44]. This can be achieved through two primary approaches. Passive targeting leverages the enhanced permeability and retention (EPR) effect, where nanoparticles passively accumulate in the leaky vasculature of inflamed and infected tissues [45]. This concentrates the therapeutic payload at the site of infection while sparing healthy tissues [46]. Alternatively, active targeting involves functionalizing the surface of nanoparticles with specific ligands, such as antibodies, peptides, or aptamers, which bind to bacterial cells or host cell receptors [47]. This provides precise and selective delivery, enhancing local antibiotic concentration and improving therapeutic outcomes [48]. This targeted delivery also improves drug stability, bioavailability and half-life, protecting the drug from enzymatic degradation [49]. Nanoparticles also demonstrate superior capabilities in overcoming biofilm-associated resistance. Their small size and tunable surface properties allow for improved penetration into dense biofilm matrices [50]. Furthermore, functionalized nanoparticles can be engineered for enhanced uptake into host cells, enabling them to effectively reach and eradicate the intracellular bacteria that are typically recalcitrant to conventional treatments [51]. Another advanced strategy involves the use of stimuli-responsive nanoparticle systems, which are designed for ‘on-demand’ drug release in response to specific environmental cues at the infection site [52]. These triggers can be physical (e.g., temperature or light), chemical (e.g., pH changes or redox potential), or biological (e.g., enzymatic activity or bacterial toxins) [53]. For example, pH-responsive systems are particularly valuable for targeting infections in acidic environments, such as the skin or gastrointestinal tract, ensuring that the therapeutic payload is released only where it is needed [54].

Nanoparticles are also revolutionizing infectious disease prevention through the development of nanovaccines. These platforms enhance vaccine efficacy by improving antigen stability, promoting efficient uptake by antigen-presenting cells (APCs) and enabling the co-delivery of antigens and adjuvants [55]. The ability to co-deliver these components in a single nanoparticle allows for the programmed modulation of immune responses, leading to more robust and durable humoral and cellular immunity [56]. This is promising approach for generating strong immune responses against challenging bacterial pathogens such as *Staphylococcus aureus* and *Mycobacterium tuberculosis* [57].

As even more sophisticated strategy is the development of antivirulence nanotoxoid vaccines. This biomimetic approach addresses a central problem in antimicrobial therapy by targeting bacterial virulence factors such as toxins, rather than the pathogen’s core survival mechanisms [58]. By focusing on disarming the bacteria, this strategy avoids imposing selective pressure for resistance. The technology employs ‘nanosponges’, which are nanoparticles coated with natural cell membranes (e.g., from red blood cells or macrophages) [59]. These membrane-coated particles act as decoys, intercepting and neutralizing pore-forming toxins before they can damage host tissues. Preclinical studies have shown that red blood cell membrane-coated NPs can completely abolish the haemolytic activity of the toxin a-hemolysin from *S. aureus* [60]. This approach is particularly elegant as it bypasses the traditional cycle of drug discovery and resistance development, representing a major conceptual leap in infectious disease therapy.

## 5. Nanoparticles in Synergistic and Combination Therapies

While the development of entirely new nanoparticle-based drugs is a long-term goal with significant regulatory hurdles, the most pragmatic and promising approach for near-term clinical impact is the use of nanoparticles in combination with existing conventional antibiotics. This synergistic strategy leverages the multi-target nature of nanoparticles to enhance the efficacy of antibiotics, effectively ‘reviving’ drugs that have become ineffective against multidrug-resistant strains [61].

The rationale for combination therapy is to create a multi-pronged attack that is far more difficult for bacteria to overcome. Nanoparticles and antibiotics can work together in several synergistic ways. Firstly, nanoparticles can increase the membrane permeability of bacterial cells [62]. By causing physical or chemical damage to the bacterial membrane, NPs create pathways that allow conventional antibiotics to enter the cell more easily, thereby increasing their intracellular concentration and efficacy. Nanoparticles can also overcome bacterial efflux pump mechanisms [63]. Many MDR bacteria use these pumps to actively expel antibiotics from the cell, a primary mechanism of resistance. Nanoparticles can either inhibit these pumps or serve as a delivery vehicle that bypasses them entirely, ensuring that the drug reaches its intracellular target [20]. This is especially relevant for intracellular pathogens that have developed resistance to existing therapies. Finally, NP-antibiotic conjugates can be more effectively taken up by bacterial cells, as the combined complex is not recognized by the traditional resistance pathways [64].

The therapeutic advantages of these synergistic combinations are manifold. They can restore the efficacy of old or previously ineffective antibiotics, expanding the therapeutical arsenal available to clinicians. Moreover, synergy allows for a lower required dosage of both the antibiotic and the nanoparticle, which is crucial for minimizing the potential toxicity and side effects associated with high-dose treatments [65]. The increasing use of computational methods, such as machine learning and genetic algorithms, is further accelerating this field by identifying highly synergistic drug-nanoparticle combinations with remarkable efficiency, such as the pairing of gold NPs with chloramphenicol [66]. This computational approach represents an important strategic direction, as it offers a more immediate and scalable path to clinical use compared to the development of a wholly new therapeutic.

Recent studies provide both in vitro and in vivo evidence supporting this synergistic effect. For instance, Thappeta et al. demonstrated that cationic polymer nanoparticles enhanced the efficacy of multiple antibiotics against MDR Gram-negative bacteria [67]. Adeniji et al. reviewed several antibiotic–nanomaterial combinations and found consistent synergy across AgNPs, AuNPs, and ZnO-based systems [61]. Moreover, Taheri-Araghi et al. reported that combining antimicrobial peptides with antibiotics markedly reduced resistance development [60]. Importantly, in vivo data from El-Khadragy et al. confirmed that AgNPs significantly enhanced the clinical efficacy of conventional therapy against Leishmania major infection [44].

## 6. Critical Challenges to Clinical Translation

A comprehensive review of the role of nanoparticles in combating AMR would be incomplete without a critical examination of the significant hurdles that must be overcome for their widespread clinical translation. While their promise is immense, the path from laboratory to patient is fraught with challenges related to bacterial adaptation, toxicity, and regulatory barriers. The assumption that bacteria cannot develop resistance to nanoparticles is not entirely accurate. While the multi-mechanistic nature of NPs makes resistance more difficult to acquire compared to single-target antibiotics, bacteria can still adapt (Table 2) [19]. Evidence suggests that bacteria can develop resistance to nanomaterials, for example, through genetic changes induced by silver nanoparticles that lead to cross-resistance to conventional antibiotics. Bacteria have also been shown to employ various defence mechanisms, including efflux pumps to expel toxic ions, surface modifications to prevent NP adhesion, chemical neutralization of released ions, and the upregulation of antioxidant responses to counteract ROS. These adaptations reflect the inherent genetic plasticity of bacteria and underscore the need for prudent application of NP-based therapies to maintain long-term efficacy [68].

Perhaps the single biggest technical hurdle to clinical adoption is the toxicity paradox. The properties that make nanoparticles so effective against bacteria, particularly their ability to generate ROS, are also the primary mechanisms of their toxicity to human cells. This creates a fine line that researchers must walk—achieving bactericidal efficacy without inducing host cell damage [69]. The toxicity of nanoparticles is a complex function of their physicochemical properties, including size, shape, surface charge, and chemical composition (Table 3). For instance, smaller particles generally exhibit higher toxicity due to their increased surface-to-volume ratio, which allows for greater interaction with biological systems. Positively charged NPs are more likely to be taken up by negatively charged human cells, leading to increased toxicity. This can lead to organ-specific damage, with NPs accumulating in and causing harm to the lungs, liver, and kidneys, potentially leading to conditions like pulmonary fibrosis [24].

Finally, the regulatory and manufacturing landscape presents a significant chasm between laboratory innovation and industrial application. The hybrid nature of nanomedicines—often combining chemical, biological, and mechanical elements—means they do not fit neatly into traditional regulatory classifications of drugs, biologics, or medical devices [69]. This creates a lack of standardized guidelines and significant ambiguity in the approval process. Furthermore, the lack of standardized synthesis protocols and manufacturing inconsistency is a major obstacle. Even small changes in the synthesis process can lead to significant variations in the physicochemical properties of nanoparticles, which, in turn, can affect their safety and efficacy [70]. Regulatory bodies require predictable and replicable data to assess health risks and benefits, something that the current state of nano-manufacturing struggles to provide.

**Table 2:** Bacterial Resistance Mechanisms against Nanoparticles.

Mechanism	Description	Example of Pathogens, Where Reported	NP Types Implicated	Ref.
Efflux pumps/metal efflux systems	Membrane-spanning proteins that actively expel toxic substances, including heavy metal ions and nanomaterials, from the bacterial cell	*E. coli*, *P. aeruginosa*	AgNPs, Cu NPs, metal ions	[71,72]
Surface modification/charge alteration (prevent NP adhesion)	Alteration of the bacterial cell membrane composition or surface charge to prevent the adhesion or penetration of nanoparticles	*S. aureus*, *E. coli*	Metallic and carbon NPs	[72,73]
Gene expression changes/stress responses	Exposure to nanoparticles induces changes in bacterial gene expression, leading to adaptive responses and even cross-resistance to conventional antibiotics	*E. coli*, *Enterococcus* spp.	AgNPs, metal oxides	[74]
Biofilm/EPS sequestration (physical barrier; adsorption of NPs)	Bacteria form a self-produced extracellular polymeric substance (EPS) matrix that acts as a physical and chemical barrier, limiting the penetration of nanoparticles and protecting embedded cells	*P. aeruginosa*, *mixed biofilms*	ZnO, SiO_2_, Ag, other NPs	[16,75]
Chemical neutralization/sequestration by secreted biomolecules	Some bacteria can chemically modify or neutralize the toxic ions released by nanoparticles before they can cause cellular damage	*E. coli*, environmental strains	AgNPs, ZnO	[15,16]
Internal sequestration/metal ion homeostasis	Bacteria can sequester internalized nanoparticles or toxic species within the cell, effectively rendering them harmless	*Pseudomonas*, metal-accumulating strains	Gold, silver, other metals	[75]
Aggregation/extracellular aggregation (e.g., flagellin-mediated aggregation)	Bacteria secrete extracellular proteins such as flagellin, which bind and induce aggregation of nanoparticles into larger, inert clusters. This reduces nanoparticle surface area, diminishes ion release and ROS generation, and prevents direct contact with the bacterial cell surface, thereby lowering antimicrobial efficacy	*E. coli*, *S. aureus*	AgNPs (citrate-coated and others)	[73,74]

**Table 3:** Factors Influencing Nanoparticle Toxicity.

Factor	How It Modifies Toxicity	NP Types Where Most Relevant	Ref.
Size	Smaller diameter increases surface area and ion release; increases cellular uptake	Metallic NPs (Ag, ZnO), metal oxides, carbon dots	[13,76]
Shape/aspect ratio	High-aspect-ratio particles (nanorods, nanotubes) show different biodistribution and mechanical damage potential	CNTs, nanorods, nanofibers	[46,59]
Surface charge (zeta potential)	Cationic NPs bind bacterial and mammalian membranes more strongly; increased host cell uptake/toxicity	Cationic polymeric NPs, functionalized metallic NPs	[56]
Composition/intrinsic material toxicity	Toxic element-based NPs (e.g., Cd) show inherent toxicity even at low dose	Cd-based QDs, some metal oxides	[20]
Protein corona	Adsorbed proteins change biodistribution, stability, and immune recognition	All NP types in biological fluids	[46]
Route of exposure/biodistribution	Inhalation, intravenous, dermal routes affect organ-specific accumulation	All NP types	[56,59]
Dose/chronic exposure	Higher dose and chronic exposure increase accumulation and toxicity risk	All NP types	[13,20]

## 7. Discussion

### 7.1. Summary of Key Findings

This systematic review reveals that nanoparticles are a potent weapon against antimicrobial resistance, utilizing a diverse array of mechanisms to combat bacterial pathogens. However, the analysis also confirms that bacteria are not passive targets. They have already developed sophisticated defence mechanisms, including efflux pumps, surface modifications, and genetic adaptations, which pose a significant threat to the long-term efficacy of these therapies. This highlights a critical need to move beyond simply demonstrating the antimicrobial properties of NPs and instead to focus on how these agents can be designed to specifically overcome bacterial resistance pathways.

### 7.2. Strengths and Limitations

The strengths of this review lie in its systematic and reproducible methodology, a comprehensive search strategy across multiple databases, and a detailed analysis of both nanoparticle-based antimicrobial mechanisms and the documented bacterial resistance pathways. The review also critically evaluates the quality of the evidence, highlighting the need for improved reporting standards in nanomedicine research.

A significant limitation of the current body of literature is the substantial heterogeneity of study designs, ranging from diverse nanoparticle formulations to varied bacterial strains and experimental conditions. A particularly pressing issue is the lack of standardized reporting on nanoparticle physicochemical properties (e.g., size, shape, surface charge, concentration) and synthesis methods. This variability complicates the interpretation and comparability of findings across studies. This observation underscores a significant gap in the field—a critical “translation gap” exists between innovative laboratory-based findings and their successful application in clinical settings. The evidence base is largely composed of in vitro and preclinical data, with a scarcity of long-term in vivo or clinical studies, which is essential to assess safety, scalability, and long-term efficacy in a therapeutic context.

### 7.3. Clinical Implications and Future Research Directions

The findings of this review have profound implications for future research and clinical practice. It is evident that the most promising avenue for nanomedicine in AMR is not the complete replacement of antibiotics but a synergistic revolution where nanoparticles act as “resistance bypass agents”. For example, the use of dendrimers to facilitate the intracellular delivery of antibiotics can circumvent efflux pump-mediated resistance, while formulations with compounds like simvastatin can disrupt the protective biofilm matrix, allowing conventional antibiotics to reach their targets. This strategic, multi-pronged approach is far more likely to yield sustained success than single-agent therapies.

Future nanoparticle design must be “resistance-informed” from the outset. Rather than solely focusing on antimicrobial lethality, researchers should prioritize targeting specific bacterial defence mechanisms. This includes developing nanoparticles that can actively disrupt the biofilm matrix, inhibit the function of efflux pumps, or block the expression of resistance-related genes. A particularly innovative and promising approach identified in the literature is the use of nanotoxoid vaccines, which target bacterial virulence factors (e.g., toxins) rather than the pathogen itself. This strategy does not impose selective pressure on bacterial survival and therefore minimizes the likelihood of resistance evolution.

## 8. Conclusions

This systematic review confirms that nanoparticles represent a powerful, multifaceted weapon in the global fight against antimicrobial resistance. Their diverse mechanisms of action offer a significant advantage over conventional antibiotics, which are often outmanoeuvred by bacterial defences. However, the emergence of bacterial resistance to nanoparticles is a real and growing threat that cannot be ignored. The path forward lies in rational, resistance-informed nanoparticle design, synergistic combination therapies, and a concerted effort to bridge the gap between innovative laboratory research and successful clinical translation. By strategically leveraging the unique properties of nanoparticles to disarm bacterial defences, and by embracing resistance-agnostic approaches, nanomedicine can play a transformative role in securing the future of infection control.

## Figures and Tables

**Table 1:** Comparative Analysis of Nanoparticles vs. Conventional Antibiotics.

Feature	Conventional Antibiotics	Nanoparticle-Based Strategies	Ref.
Mechanism of action	Single-target biochemical pathways	Diverse, multi-target mechanisms (ROS generation, membrane disruption, ion release, physical disruption)	[8,15]
Biofilm efficacy	Poor penetration; reduced activity in biofilm matrix	Better penetration (size/surface tuning), active biofilm disruption strategies reported	[16,17]
Resistance development	Rapid selection for single-target resistance	Less likely but documented adaptation/tolerance (e.g., aggregation, efflux, cross-resistance)	[18,19]
Tunability	Fixed chemical scaffold per drug	Highly tunable (size, shape, surface chemistry, coatings, stimuli-responsive release)	[6,8]
Clinical application/translation	Established; many approved antibiotics	Emerging; several preclinical and some clinical formulations; safety & regulatory hurdles remain	[8,20]

## Data Availability

No new data were created or analyzed in this study.
